# circHtra1/miR-3960/GRB10 Axis Promotes Neuronal Loss and Immune Deficiency in Traumatic Brain Injury

**DOI:** 10.1155/2022/3522492

**Published:** 2022-05-06

**Authors:** Ping Zheng, Liang Shu, Dabin Ren, Zhucai Kuang, Yisong Zhang, Jian Wan

**Affiliations:** ^1^Department of Neurosurgery, Shanghai Pudong New Area People's Hospital, China; ^2^Department of Neurology, Shanghai Ninth People's Hospital, China; ^3^Department of Emergency Medicine, Shanghai Pudong New Area People's Hospital, China

## Abstract

Circular RNAs (circRNAs) are abundant in the brain and contribute to central nervous system diseases; however, the exact roles of circRNAs in human traumatic brain injury (TBI) have not been established. In this study, we used a competing endogenous RNA (ceRNA) chipset as well as *in vitro* and *in vivo* assays to characterize differentially expressed circRNAs in TBI. We detected 3035 differentially expressed circRNAs in the severe TBI group, 2362 in the moderate group, and 433 in the mild group. A ceRNA network was constructed. The circRNA has_circ_0020269 (circHtra1) was significantly upregulated after brain insults and was correlated with the severity of injury. circHtra1 inhibited cell proliferation and promoted apoptosis, and its knockdown reversed these effects. Further analyses revealed that circHtra1 functions as a miR-3960 sponge and increases the expression of *GRB10*, which is involved in NK cell infiltration after TBI. circHtra1 was identified as a target of the IGF-1/ADAR1 axis. Reduced expression of ADAR1 (involved in A-to-I editing) after brain insults upregulated circHtra1. Our results show that circHtra1 promotes neuronal loss by sponging miR-3960 and regulating GRB10 and apoptosis during brain insults. In addition, A-to-I editing could regulate circRNA expression profiles after TBI, and circHtra1 is a potential therapeutic target.

## 1. Introduction

Traumatic brain injury (TBI) is associated with neurodegeneration, cognitive impairment, and psychiatric disorders, representing an enormous burden on modern society [1]. However, exact molecular and pathological changes in TBI are not clear. Noncoding RNAs have recently become novel targets for both mechanistic and therapeutic studies [2, 3]. However, few studies have evaluated tissue- or developmental stage-specific expression patterns of circular RNAs (circRNAs) and their regulatory effects in TBI. circRNAs are highly expressed in the brain, are specifically related to neuronal and synaptic function, and have been identified as independent biomarkers [4]. This type of RNA constitutes a large class of posttranscriptional regulators, some of which can act as ceRNAs by inhibiting miRNAs in the brain [5]. For instance, ciRS-7 acts as a miR-7 sponge, leading to the increased expression of miR-7 target genes, particularly in neocortical neurons and tumor cells as well [6].

Recently, Jiang et al. found a series of circRNAs widely distributed in the cortex of mice with TBI [7]. However, studies of circRNA profiles as well as their diagnostic and therapeutic value in human TBI are limited. Accordingly, we used a competing endogenous RNA (ceRNA) chipset to evaluate circRNAs and target genes in blood samples from humans with TBI and constructed a ceRNA network. A new circRNA, circHtra1, was identified, and its relationship with clinical features was assessed in both *in vitro* and *in vivo* brain injury models. Our findings highlight the important role of the circHtra1-miR-3960-GRB10 axis and its potential therapeutic value for TBI.

## 2. Results

### 2.1. Differentially Expressed circRNAs in the Blood of Humans with TBI

**Figure 1:**
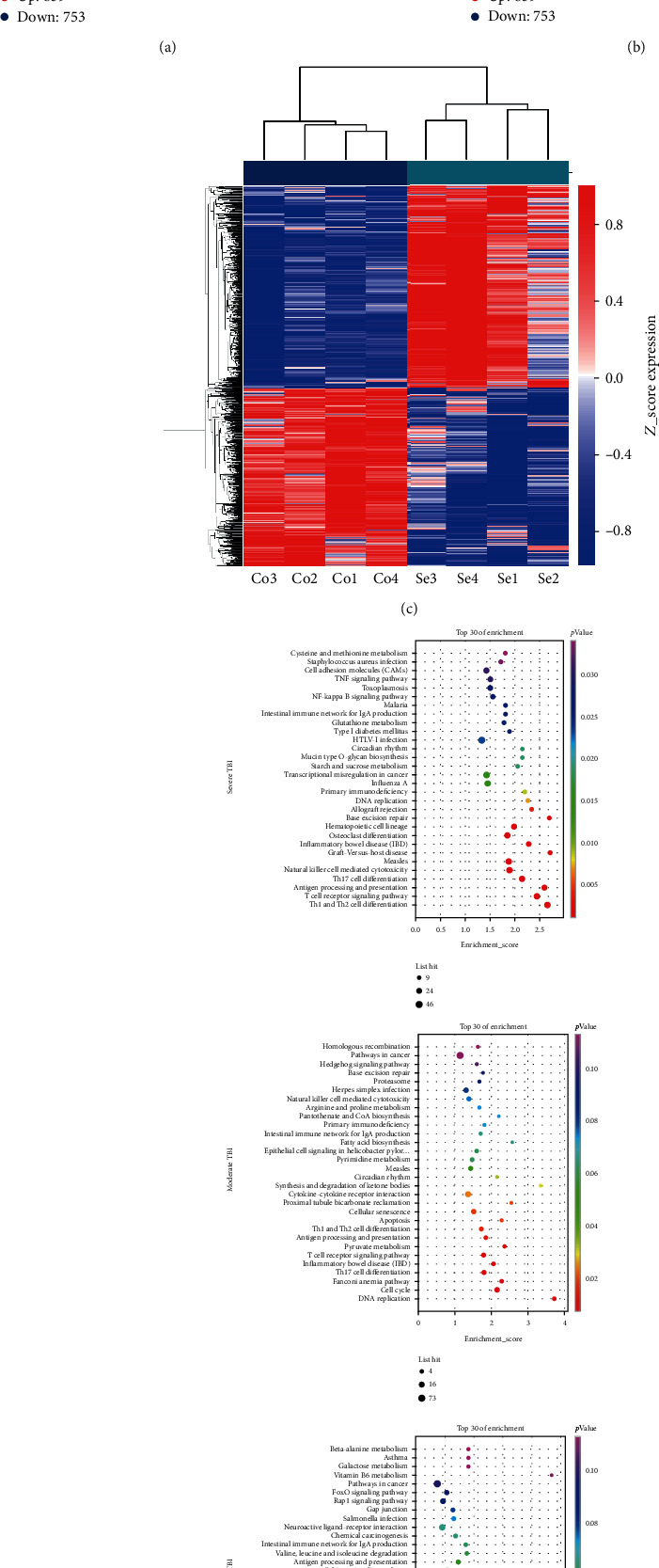
Volcano plots, clustering analysis, and functional enrichment analysis of circRNAs and target genes. (a, b) Volcano and scatter plots revealed that 3035 circRNAs were differentially expressed between the severe group and the control group, and four severe TBI groups clustered together. (c) Top 30 KEGG pathway results visualized by bubble plots for TBI groups. (d) Upper panel: related target genes in severe TBI. Middle panel: related target genes in moderate TBI. Lower panel: related target genes in mild TBI.

Differentially expressed circRNAs between patients with severe, moderate, and mild TBI and healthy controls were identified. As visualized by a volcano plot, there were 1612 differentially expressed circRNAs between the severe group and the healthy control group (*P* < 0.05, fold change > 2) (Figure 1), including 859 upregulated circRNAs and 753 downregulated circRNAs. The five most significantly upregulated circRNAs were hsa_circ_0020273, hsa_circ_0093014, HSA_CIRCpedia_152345, hsa_circ_0020269, and hsa_circ_0064339, with logFC values of 3.7–4.3. The five most significantly downregulated circRNAs were hsa_circ_0061796, hsa_circ_0129469, HSA_CIRCpedia_35578, hsa_circ_0070423, and hsa_circ_0116097, with logFC values ranging from -3.9 to -3.1 (Table S3).

In the moderate TBI group, 287 circRNAs were upregulated and 370 circRNAs were downregulated. In the mild group, 41 circRNAs were upregulated, and 67 circRNAs were downregulated. These results suggest an obvious trend of increase in the total number of altered circRNAs with increase in the severity of brain injury. Altered circRNAs were clearly separated into two clusters in a heatmap, indicating that samples had good intragroup consistency, and circRNAs expressed in the TBI group were significantly different from those in the healthy control group (Figure 1(c)).

### 2.2. GO and KEGG Analyses

A GO enrichment analysis of target genes of differentially expressed circRNAs was performed to evaluate alterations in molecular functions (MF), biological processes (BP), and cellular components (CC). The target genes of circRNAs in the severe TBI group were mainly enriched for the adaptive immune response, neutrophil degranulation, and defense response to virus (in the BP category, Figure 1(d)).

We also performed a KEGG pathway enrichment analysis and generated a bubble chart of the top 30 pathways related to altered target genes, including Th1 and Th2 cell differentiation, T cell receptor signaling pathway, and antigen processing and presentation (Figure 1). Target genes were mostly involved in T cell pathways and immunity in both moderate and severe TBI. In particular, the immune response was within the top three most highly enriched pathways in both the severe TBI and moderate TBI groups.

### 2.3. Establishment of a ceRNA Network

Using Cytoscape, relationships between individual circRNAs, miRNAs, and mRNAs were determined based on Pearson's coefficient coefficients. After rigorous selection, a circRNA–mRNA coexpression network was constructed based on the 200 most significant circRNA-associated ceRNA pairs. Several ceRNA pairs with high ceRNA scores and energy values were chosen to form a circRNA–miRNA–mRNA network (Figure S1). In particular, circRNA_0020269, CIRCpedia_36273, circRNA_0116394, CIRCpedia_152529, and circRNA_0099010 pairs showed competitive binding to dozens of miRNAs, such as hsa-miR-10400-5p, and hsa-miR-3960.

To evaluate whether the circRNAs could impact pathways by acting as ceRNAs, one core circRNA, circRNA_0020269, was further investigated. A circRNA–miRNA–mRNA interaction network derived from circRNA_0020269 and five miRNAs was analyzed further (Figure S1). The circRNA_0020269 was predicted to act as a ceRNA for four miRNAs.

### 2.4. si-circHtra1 (circRNA_0020269) Facilitates Neuronal Maturation and Proliferation

**Figure 2:**
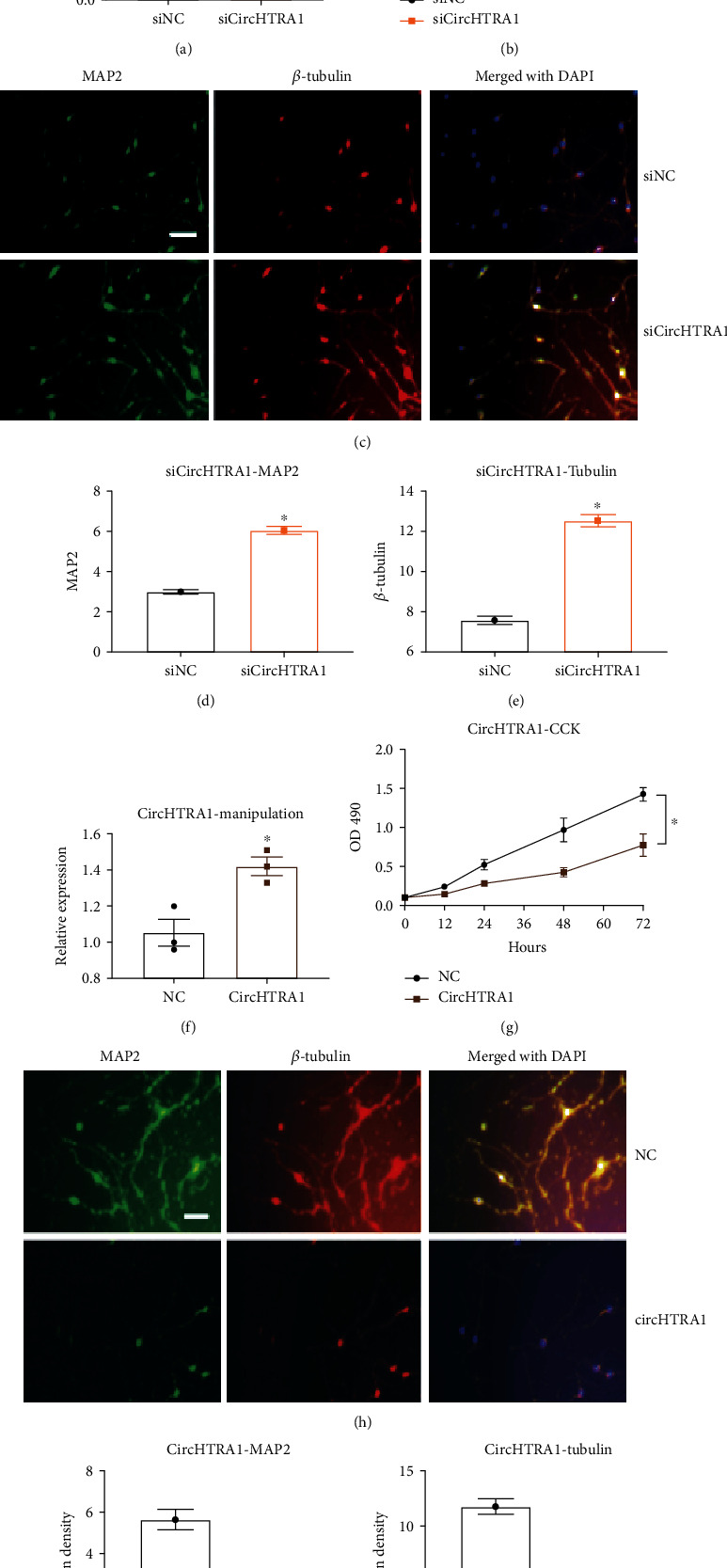
Effects of circHtra1 on neuronal proliferation. (a) Expression of circHtra1 in si-circ was verified. (b**)** After primary cortical neurons were transfected with si-NC or si-circHtra1, a CCK-8 assay was applied to assess cell proliferation. (c) Effect of si-circHtra1 on neuronal maturation (green: MAP2; red: *β*-tubulin, merged with DAPI). (d, e) Quantification of mean densities of MAP2 (*t* = 9.496, *df* = 4) and *β*-tubulin (*t* = 12.14, *df* = 4). Scale bar represents 200 *μ*m. ^∗^*P* < 0.05 (compared to the si-NC group); effects of circHtra1 on neuronal proliferation. (f) Verification of circHtra1 overexpression. (g) Primary cortical neurons were transfected with NC or circHtra1. (h) Effect of circHtra1 on neuronal proliferation (green: MAP2; red: *β*-tubulin, merged with DAPI). (i, j) Quantification of mean densities of MAP2 (*t* = 7.964, *df* = 4) and *β*-tubulin (*t* = 9.245, *df* = 4). Scale bar represents 200 *μ*m. ^∗^*P* < 0.05 (compared to the NC group).

Because plasma circHtra1 was upregulated in TBI, we next investigated its effect on primary cultured neurons. si-circHtra1 was used to knock down the expression of circHtra1, using si-NC as a control. As determined by CCK-8 assays, primary cultured neurons transfected with si-circHtra1 showed higher cell proliferation than that in the control (Figures 2(a) and 2(b)). Similarly, transfection with si-circHtra1 significantly increased MAP2 and *β*-tubulin expression in cell culture (*P* < 0.05, Figures 2(c)–2(e)). In contrast, circHtra1 overexpression inhibited CCK-8 expression in primary neurons and reduced MAP2 and *β*-tubulin levels (Figures 2(f)–2(j)), while si-circHtra1 or circHtra1 overexpression did not alter *Htra1* mRNA level (Figure S1 A&B).

### 2.5. Circular RNA circHtra1 Facilitates Neuronal Death via the miR-3960/GRB10 Axis

**Figure 3:**
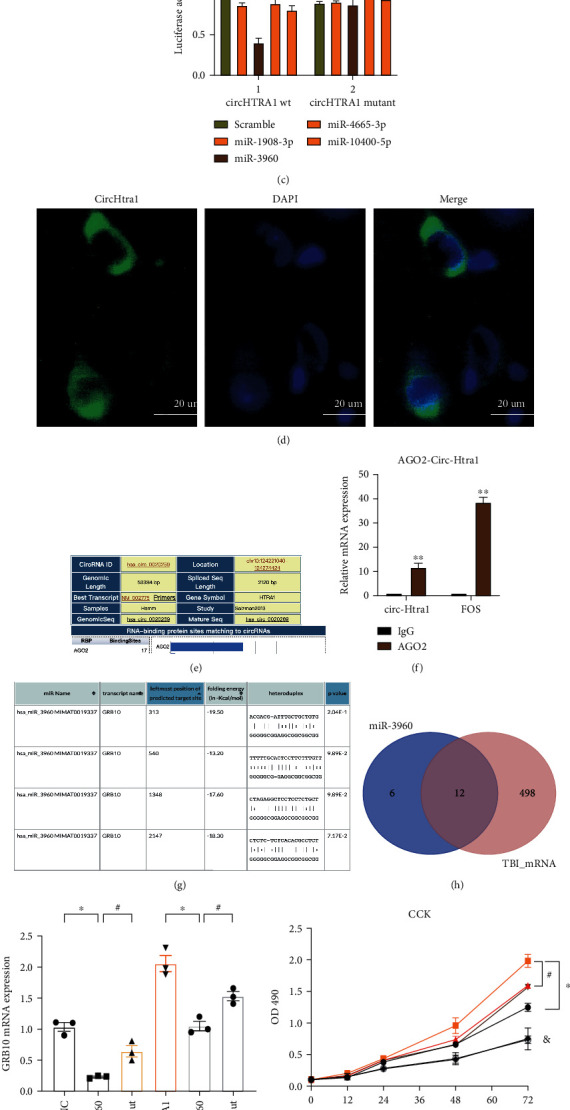
circHtra1 binds to miR-3960 and GRB10 in neurons. (a) Based on the interactome prediction, four miRNAs were predicted to bind to circHtra1. (b) The complementary site between circHtra1 and miR-3960 is shown. (c) Primary cultured neurons were cotransfected with each miRNA mimic and the wild-type circHtra1 reporter or mutant circHtra1 reporter. The *y*-axis shows the luciferase intensity. The scrambled mimic was used as a control. (d) RNA FISH for circHtra1 detection in primary cultured neurons. Nuclei were stained with DAPI (blue), and circHtra1 appears green. circHtra1 was dominantly located in the cytoplasm. Scale bars represent 20 mm. (e, f) circHtra1 is predicted to interact with AGO2 (a miRNA binding protein), as supported by a RIP assay. (g, h) miR-3960 could bind to Htra1 in three positions, as predicted by miRanda. (i) After primary cultured neurons were transfected with NC, miR-3960, miR mutant, circHtra1, circHtra1+miR-3960, and circHtra1+miR mutant, mRNA levels of *htra1* were assessed in primary cultured neurons *t* = 11.57, *df* = 4; *t* = 4.503, *df* = 4; *t* = 6.680, *df* = 4; and *t* = 4.563, *df* = 4, respectively. (j) After transfection with si-NC, si-circHtra1, or si-circHtra1+miR-3960 antagonist, si-circHtra1+Htra1, miR-3960 antagonist and Htra1, CCK-8 levels were assessed. ^∗^^, #, &^*P* < 0.05.

To determine whether circHtra1 functions as a miRNA sponge, as suggested by the ceRNA network analysis, we assessed the sequence of *circHtra1* using miRanda and circBase and identified four candidate miRNAs (miR-1908, miR-3960, miR-4665-3p, and miR-10400-5p; Figures 3(a)–3(c)). The cellular location of circHtra1 was investigated by fluorescence in situ hybridization (FISH), revealing dominant expression in the cytoplasm (Figure 3(d)). AGO2 is essential for miRNA silencing of gene expression by forming the RNA-induced silencing complex (RISC). We predicted that AGO2 could bind to circRNAs and miRNAs (based on predicted relationships between circHtra1 and AGO2 by CircInteractome). Accordingly, we performed an RNA immunoprecipitation (RIP) assay to pull down RNA transcripts that bind to AGO2 in cultured neurons. Indeed, endogenous circHtra1 was efficiently pulled down by anti-Ago2 (Figures 3(e) and 3(f)). To further test whether circHtra1 could sponge miRNAs, an miRNA pull-down assay was performed using biotin-coupled miRNA mimics (miR-1908, miR-3960, miR-4665-3p, and miR-10400-5p). Interestingly, circHtra1 was only efficiently enriched by miR-3960 but not by the other three miRNAs (Figures 3(b) and 3(c)). To confirm the interaction, we performed a luciferase assay for miR-3960 and circHtra1. Luciferase intensity decreased after cotransfection with the wild-type (WT) luciferase reporter and miR-3960 mimics, while the mutant luciferase reporter did not have the same effect (Figure 3(c)).

### 2.6. GRB10 Is a Direct Target of miR-3960 and Is Positively Regulated by circHtra1

Our results demonstrated that circHtra1 can bind directly to miR-3960 and act as an mRNA sponge. We next identified miR-3960 target genes. Using RNA22 v2, *GRB10* was a predicted target of miR-3960, and the potential binding sites are listed in Figure 3(g). Candidate target genes were selected based on bioinformatics predictions and mRNA coexpression in TBI. To investigate the miR-3960 target genes in neurons and their correlations with circHtra1 levels, cortical neurons were transfected with NC mimics, miR-3960 mimics, mutant miR-3960, or circHtra1 or cotransfected with circHtra1 and miR-3960 mimics or its mutant form. Overexpression of miR-3960 (but not mutant miR-3960) decreased *GRB10* mRNA level; thus, *GRB10* was a target of miR-3960 in neurons (Figure 3(h)). Furthermore, circHtra1 also increased the mRNA level of *GRB10*, and circHtra1-induced upregulation of *GRB10* was attenuated by miR-3960 mimics (Figure 3(i)). Additionally, a CCK-8 test showed that si-circHtra1 promoted neuronal proliferation, which was blocked by an miR-3960 antagonist or GRB10 overexpression. When primary cultured neurons were transfected with si-NC, si-circHtra1, or si-circHtra1+miR-3960 antagonist and miR-3960 antagonist, cell proliferation was significantly lower in the si-circHtra1+ miR-3960 antagonist group than in the si-circHtra1 group (*P* < 0.05, Figure 3(j)), and this effect was partially blocked by treatment with mutant miR-3960. These results indicate that circHtra1 promotes cell death by sponging miR-3960.

### 2.7. si-circHtra1 Has Neuroprotective Effects in TBI

Using the Human Assembly (GRCh37/hg19) on the UCSC Genome Browser, we searched chr10:124221040–124274424 (53385 bp) and found that circHtra1 was conserved in rhesus macaques, mice, dogs, and elephants (Figure S2A). Therefore, we used a mouse model to explore the effect of circHtra1 *in vivo*. AAVs transfected with si-NC or si-circHtra1 and mixed with miR-3960 antagomir or NC antagomir were injected subcutaneously into lateral ventricles of mice with TBI. HE staining showed that knocking down the expression of circHtra1 markedly decreased the brain injury in both the cortex and hippocampus *in vivo* (Figure S2B). We performed behavioral tests, including analyses of the neurological severity score (NSS) and paw grasping ability, to assess the motor function of mice with TBI. si-circHtra1 could reduce motor dysfunction in mice with TBI, as reflected by a lower NSS and grasping score. The neuroprotective effect of si-circHtra1 was significantly blocked in the si-NC and si-circ mixed with miR-3960 antagomir groups (*P* < 0.05, Figure S2C). Taken together, these findings indicate that circHtra1 promoted neuronal loss and motor impairment in brain insults *in vivo*.

### 2.8. Downregulation of circHtra1 Reduces the Number of Annexin-Positive Cells, GRB10, and Cleaved Caspase-3 in a Mouse Model of TBI

**Figure 4:**
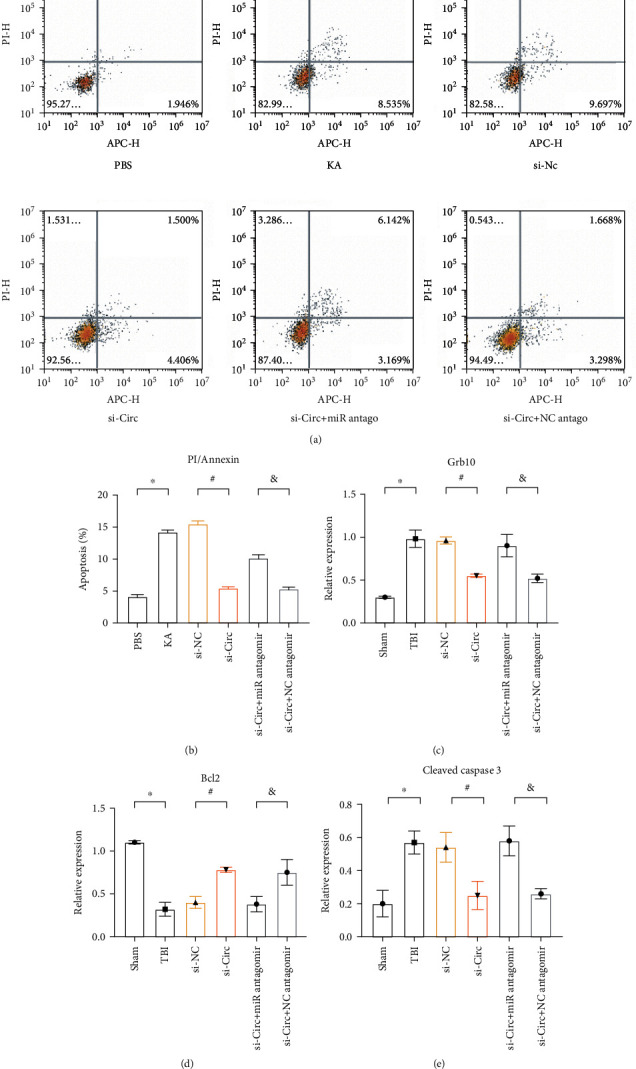
si-circHtra1 inhibits apoptosis in TBI. (a) Apoptosis of neurons in primary cortical neurons (expressed as percentages). (b) Apoptosis quantification in each group. (c–f) Protein levels of GRB10, cleaved caspase-3, and Bcl-2 determined by ELISA *n* = 3. Compared with the sham group, ^∗^*P* < 0.05; compared with the si-NC group, ^#^*P* < 0.05; and compared with the siRNA+miR antagomir group, ^&^*P* < 0.05.

Both Htra1 and GRB10 affect the Wnt and *β*-catenin pathways to regulate apoptosis and neurodegeneration. [8] We investigated the effect of circHtra1 on apoptotic markers. A PI/Annexin assay was performed to assess apoptosis *in vitro*. As shown in Figures 4(a) and 4(b), the proportion of Annexin+ cells in the KA group was significantly higher than that in the PBS group (*P* < 0.05). There was no obvious difference in Annexin+ cells between the KA group and siRNA-NC group (*P* > 0.05). The number of Annexin-positive cells in the si-Circ group was lower than that in the siRNA-NC group, and this reduction was partially blocked by the miR-3960 antagomir. The rate of apoptosis in the si-circ+NC antagomir group was lower than that in the si-circ+miR-3960 antagomir group (*P* < 0.05). We further separate the early and late apoptosis between different groups (Figure S1). We found the si-circ is able to prevent both early and late apoptosis after TBI; however, si-circ combined with miR antagomir could partly block the effects of si-circ on early apoptosis.

Expression levels of GRB10, cleaved caspase-3, and BCL-2 in the ipsilateral cortex of mice with TBI were further confirmed by ELISA. As shown in Figures 4(c)–4(e), GRB10 and cleaved caspase-3 levels were elevated in TBI, with reduced BCL-2 expression compared with those in control mice (*P* < 0.05). There were no differences in the expression levels of GRB10, cleaved caspase-3, and BCL-2 among the TBI, si-NC, and si-circ+miR-3960 antagomir groups (*P* > 0.05). GRB10 and cleaved caspase-3 levels were lower, and BCL-2 levels were higher in the si-circHtra1 group than those in the negative control group (*P* < 0.05). Compared with levels in the si-circ+miR-3960 antagomir group, GRB10 and cleaved caspase-3 expression levels were lower in the si-circ+NC antagomir group, while BCL-2 levels were higher (*P* < 0.05).

### 2.9. IGF-1 Reduces circHtra1 via ADAR1

IGF-1 has been shown to reduce the HtrA1 expression by enhancing its protease susceptibility [9]. However, the exact effects of IGF-1 on Htra1 and circRNA profiles after brain insults remain unclear. Since astrocytic IGF-1 has a neuroprotective effect [10], we further evaluated the effects of IGF-1 on circHtra1, ADAR1, and GRB10. We have recently demonstrated that IGF-1 could regulate ADAR1 expression in excitotoxicity [11]. After treatment with KA, neurons have reduced ADAR1 expression and higher calcium loads, leading to neuronal death [11]. Coculture with astrocytes can reverse this process in an IGF-1-dependent manner, as an IGF-1R antagonist could block the effect of IGF-1 on ADAR1 expression. ADAR1 also influences the expression of circRNAs [4]. Therefore, we postulated that IGF-1 has an effect on both circHtra1 and GRB10 via ADAR1.

**Figure 5:**
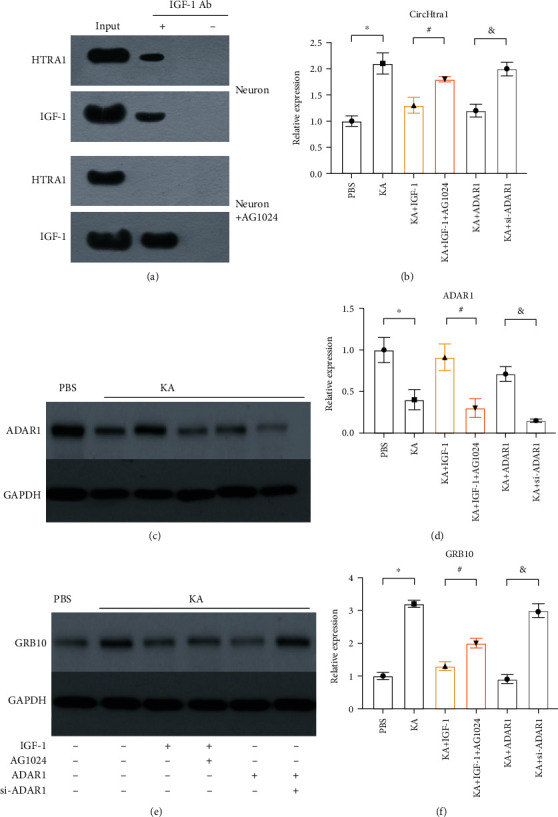
IGF-1 reduces circHtra1 via ADAR1. (a) IGF-1 is immunoprecipitated with htra1 in HEK cells, and this effect is blocked by AG1024. (b) circHtra1 expression in neurons treated with KA, IGF-1, ADAR1, and their inhibitors (*t* = 4.919, 3.162, and 8.503, respectively, *df* = 4). (c, d) Expression of ADAR1 in neurons treated with KA, IGF-1, ADAR1, and their inhibitors (*t* = 3.123, 3.142, and 6.074, respectively, *df* = 4). (e, f) Expression of GRB10 in neurons treated with KA, IGF-1, ADAR1, and their inhibitors (*t* = 11.242, 2.162, and 7.230, respectively, *df* = 4). ^∗^^, #, &^*P* < 0.05.

First, we confirmed the interaction between IGF-1 and Htra1 by Co-IP. Consistent with previous findings [9], we found that Htra1 could be immunoprecipitated by IGF-1 in HEK cells (Figure 5(a)), and this effect could be blocked by AG1024 (an IGF-1R antagonist, which has a much higher binding affinity with IGF-1). We have previously shown that KA treatment could reduce ADAR1 expression via IGF-1. In this study, IGF-1 increased ADAR1 expression after KA, and this was reversed by AG1024 (Figures 5(c) and 5(d)). Next, we evaluated the effect of IGF-1 on circHtra1. We found that KA increased the expression of circHtra1, and both IGF-1 and ADAR1 reduced its expression levels; these effects were also blocked by AG1024 or ADAR1 siRNA (Figure 5(b)). To verify the sponge effect of circHtra1, we further looked at the effects of IGF-1 and ADAR1 on GRB10 expression. Again, we found that KA increased GRB10 expression, and both IGF-1 and ADAR1 reduced GRB10 expression; these effects were also blocked by AG1024 or ADAR1 siRNA (Figures 5(e) and 5(f)). Accordingly, the effect of IGF-1 on GRB10 expression was consistent with the results of our previous chipset analysis of the PI3K-Akt pathway [10]. To further confirm the effect of ADAR1 on the expression of Grb10, we integrated the single-cell sequencing data from GEO which showed the expression of Grb10 and Htra1 were dominantly in stromal cells (Figure S3).

### 2.10. Clinical Significance of circHtra1 in TBI

Next, we investigated circRNA expression in TBI. We identified the top 10 most highly increased circRNAs and found that circHtra1 expression increased with TBI severity (Figure S3A&B). We further evaluated Htra1 and circHtra1 expression levels in patients with TBI and found that both increased with the severity of injury (Figure S3C&E). Htra1 expression in TBI was further verified using GSE data (GDS4911/1386884_at), wherein Htra1 expression increased after 12 hours of brain injury and returned to baseline levels at 48 hours in a mouse model of TBI (Figure S3D). Furthermore, circHtra1 and Htra1 expression levels were positively correlated with each other (*r*^2^ = 0.37496, *P* = 0.01; Figure S3F). These results indicated that circHtra1 is a biomarker for TBI.

### 2.11. GRB10 And NK Cell Immune Infiltration in TBI

**Figure 6:**
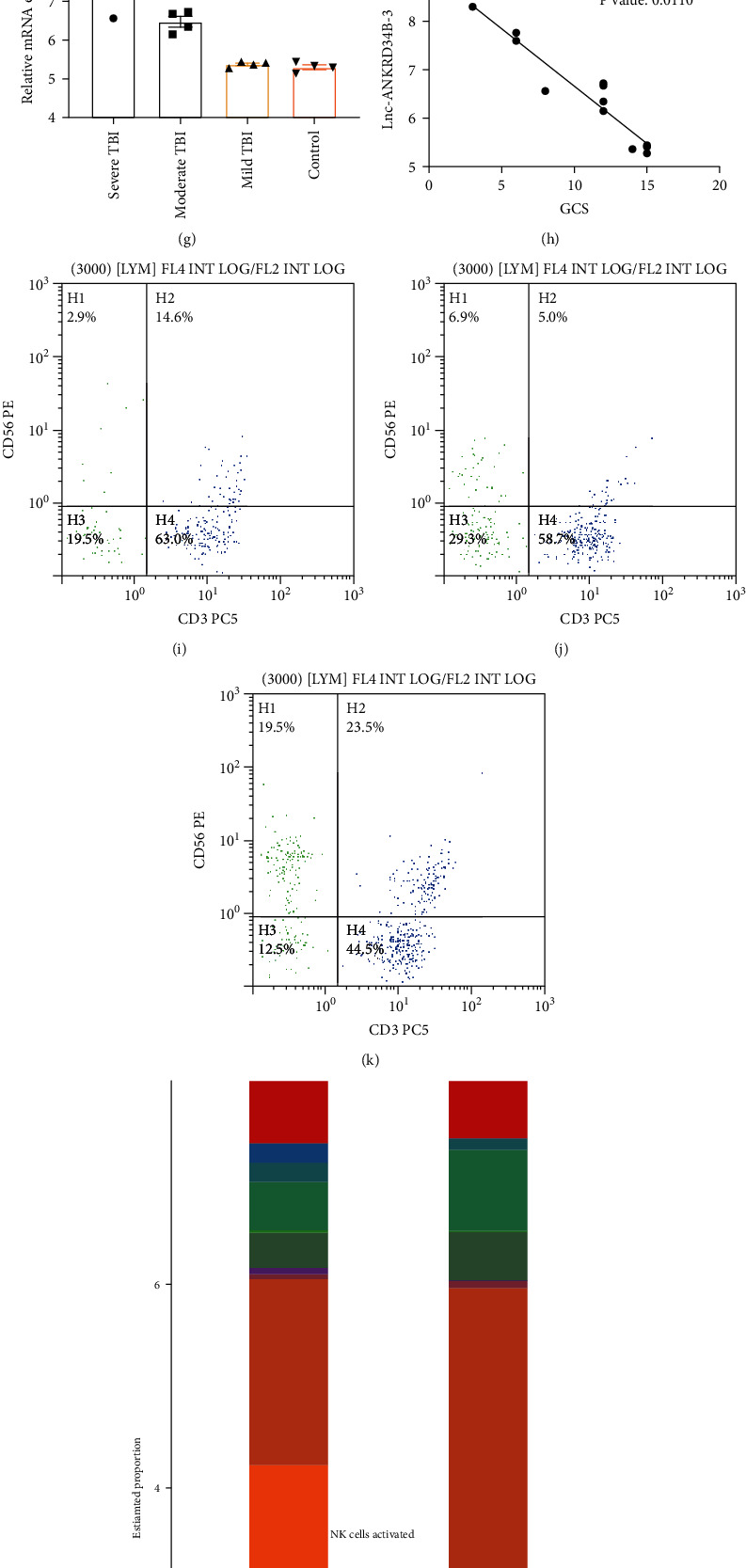
Plasma NK cell reduction in TBI. (a, b) CIBERSORT and QuanTIseq were used to assess immune infiltration in TBI. (c, d) Quantification of results shown in panels (a) and (b), showing that the percentage of NK cells decreases in moderate or severe TBI. (e, f) The percentage of NK cells is lower in severe TBI than in the mild group and is positively correlated with plasma NK (%). (g, h) The expression of GRB10 in TBI, *F*(3, 12) = 16.839 and its correlation with GCS. (i–k) The relative plasma NK ratio (reflected by CD3- CD56+) in severe TBI (i); moderate TBI (j), and mild TBI (k). (l) The CIBERSORT method shows the immune cells distribution in plasma of TBI patients and healthy controls. (m, n) The cell communication in the blood from sc-seq data in healthy controls (m) and TBI patients (n).

In our GO and KEGG pathway enrichment analyses, the immune response was the top altered pathway in moderate and severe TBI, and it has recently been reported that impaired NK cells in patients with TBI are correlated with the severity of injury [12]. We first evaluated immune infiltration in the plasma of patients with TBI based on our ceRNA chipset using CIBERSORT and QUANTISEQ (Figures 6(a) and 6(b)). We found that the NK cell percentage was lower in moderate and severe TBI than in mild cases Figures 6(c) and 6(d)). Furthermore, more severe TBI cases corresponded with a much lower NK percentage than those in the mild and moderate groups (*P* < 0.05). Plasma NK cells are positively associated with the GCS score (*R*^2^ = 0.4251, *P* < 0.01, Figures 6(e) and 6(f)) [12]. GRB10 regulates NK cells [13]. In both severe and moderate TBI, GRB10 expression levels were much higher than those in mild TBI and in the control group. Importantly, GRB10 expression was negatively correlated with the GCS score (*R*^2^ = 0.4251, *P* = 0.01; Figures 6(g) and 6(h)). Furthermore, we performed FACS analyses to evaluate the proportion of NK cells (CD56-positive and CD3-negative, upper-left quadrants of Figures 6(i)–6(k)). As the severity of brain injury increased, the population of NK cells in plasma of patients with TBI decreased. This can also be reflected by the CIBERSORT result (both numbers of resting NK and activated NK cells were reduced in TBI compared to the healthy controls, Figure 6(l)). We further applied the sc-seq in TBI patients plasma and showed that consistent with the decreased NK cells in TBI, the cell interaction between NK and monocytes was reduced as well (Figures 6(m) and 6(n)).

## 3. Discussion

Several recent studies have established the important roles of circRNAs in various central nervous system (CNS) diseases, such as Alzheimer's disease (AD), Parkinson's disease (PD), ischemic brain injury, and neurotoxicity [3, 5, 14]. They may exert critical biological functions as microRNA (sponges), or by regulating protein function. However, the exact role of circRNAs in TBI has not been deeply determined. We therefore characterized circRNA expression profiles in human TBI by a chipset analysis. We found that the total number of altered circRNAs increased as the severity of brain injury increased, indicating that gene editing is more highly impaired in severe TBI.

### 3.1. circHtra1 Is a Biomarker for TBI

In GO and KEGG functional enrichment analyses, target genes of altered circRNAs were highly enriched in the cytosol and were related to the inflammatory response and innate immunity (which may reflect their sponging function). According to the KEGG pathway analysis of the host genes for circRNAs in our study, the most enriched pathways were Th1 and Th2 cell differentiation, T cell receptor signaling pathway, and antigen processing and presentation, suggesting that an immune deficiency is critically involved in TBI.

Previous studies have focused on the circRNA profiles in mouse cortex after TBI, and up to now, almost no studies have investigated the circRNA expressions in human TBI. The five most highly upregulated circRNAs in our study were hsa_circ_0020273, hsa_circ_0093014, HSA_CIRCpedia_152345, hsa_circ_0020269, and hsa_circ_0064339. Interestingly, three of these were produced from Htra1. Further analyses revealed that circHtra1 is a promising biomarker for the severity of TBI. The circHtra1 expression level was remarkably higher after brain insults *in vitro* and *in vivo*, and its upregulation was positively correlated with Htra1 expression and the GCS score in TBI. In addition, the knockdown or overexpression of circHtra1 significantly reduced or facilitated cell loss in primary cultured neurons. With respect to the underlying mechanism, circHtra1 promoted neuronal loss by sponging miR-3960, thereby increasing GRB10 expression. Considering the stable circular structure and enrichment in the CNS, circHtra1 is a potential therapeutic target for TBI.

Of note, *htra1*, which has an IGF domain, was predicted to be competitively regulated by circRNA_0020269. The knockdown *of HtrA1* activates *PI3K*/*Akt* signaling in A549 cells, which indicates that Htra1 might be a downstream of IGF-1 signaling [15]. *In vivo* studies have also shown that the knockdown of *HtrA1* promotes tumorigenesis [15]. *HtrA1* also reduces Wnt signaling by binding to *β*-catenin and decreases the rate of cell proliferation [8]. Consistent with previous results, our Annexin and PI staining and analyses of apoptosis markers revealed that circHtra1 knockdown efficiently inhibited apoptosis, including both early and late stage of apoptosis and this effect was partially blocked by miR-3960.

### 3.2. circHtra1 Is Affected by A-to-I Editing in TBI

A-to-I editing is increased during brain development [16]. In addition, the RNA editor ADAR1 could regulate neural fate and the expression levels of circRNAs in the CNS [4]. However, the regulating role of ADAR1 on circRNAs after brain injury has not been investigated previously. We therefore studied the role of ADAR1 in the biogenesis of neuronal circRNAs and found that increased circHtra1 after brain insults corresponded with reduced ADAR1 expression. This is consistent with our previous *in vitro* assays showing that excitotoxic injury reduced ADAR1 expression and affected calcium hemostasis. Astrocytic IGF-1 could reverse these pathologies. We further demonstrated that IGF-1 regulates circHtra1 expression (Figure S4A-D). However, the underlying mechanisms are unclear, and the regulatory effects might be mediated by the effects of IGF-1 on exosome release.

In both *in vitro* and *in vivo* analyses, we found that si-circHtra1 has a neuroprotective role, as evidenced by increased neuronal proliferation and improved motor function in a mouse model of TBI. RNAi delivery has been used as a therapeutic strategy in brain insults; however, circRNA interference has rarely been evaluated in clinical studies. Considering the stable structure and expression of circRNAs in the brain, these could become potential targets for clinical applications. Although circRNAs act mostly as miRNA sponges and adjust the expression of downstream mRNAs, they also bind directly to proteins or are translated into peptides [17]. Therefore, our understanding of the functions of circHtra1 could be enhanced by further RNA purification- (CHIRP-) sequencing or RIP-sequencing analyses.

### 3.3. circHtra1 Is Associated with Immune Deficiency in TBI

Inflammation and the immune response are vital mechanisms in secondary brain injury [18, 19]. Htra1 promotes inflammation and macrophage infiltration [20]. NK cells, another important immune cell subset, are rapidly recruited and promote recovery after TBI, leading to reduction in NK cell numbers in the peripheral blood [12]. Recent evidence suggests, however, that NK cells are detrimental after hemorrhagic injury [21]. The reason for these contradictory functions of NK cells remains unclear. In addition, NK cell-mediated cytotoxicity deserves further attention, as it is associated with specific circRNAs that are rarely detected in non-TBI samples. NK cells are thought to be the first line of defense for immune monitoring and exert a critical role in anti-infection therapy. circRNAs contribute to the immune response by promoting NK cell activity and upregulating NK-mediated immune responses. For example, circARSP91 enhances the cytotoxicity of NK cells in liver cancer cells [22]. Furthermore, recent research has demonstrated that hypoxia-triggered circ-0000977 inhibition promotes the killing effect of NK cells by regulating hypoxia-inducible factor 1-alpha (HIF1*α*). This axis regulates the HIF1*α*-mediated immune escape of PC cells by NK cell activity [23]. However, the direct relationship between circRNAs and NK cells and the interaction between cancer cells and NK cells have not been evaluated. Similarly, we did not study the relationship between NK cells and neurons directly. Future studies of the mechanisms by which circRNAs modulate NK cell activity are needed for the development of strategies to mediate the immune response via NK cells.

Recently, Deng et al. found that plasma GRB10 levels are correlated with NK cell counts and identified GRB10 and E2F3 as biomarkers for osteoarthritis and their association with immune infiltration [13]. NK cell alterations have also been reported in TBI; the percentage of NK cells in the peripheral blood is correlated with GCS and Glasgow Outcomes Scores (GOS) in patients with TBI [12]. These findings are consistent with those of our study, indicating that GRB10 may be involved in NK cell reduction peripherally in TBI events. The mechanisms underlying NK cell reduction and its biological consequences remain to be elucidated. Several factors could result in alterations of NK cells in TBI. First, NK cells might penetrate the damaged blood–brain barrier, thereby reducing their expression peripherally and entering the brain. Alternatively, increased susceptibility to infection could be due to immunodepression in patients with TBI, consistent with our results for the percentage of peripheral NK cells in TBI. The exact relationship between GRB10 and NK cells, among other immune cells (i.e., Th1 or Th2), also has a critically important role in the immune response after TBI, requiring further studies. It is also necessary to study the crosstalk between neurons and NK cells to investigate their interaction effects in CNS.

Some limitations of this work need to be addressed in future studies. First, the sample size was relatively limited (12 patients with TBI and 4 healthy controls). Although we were able to obtain the positive results from such experiments, as a biomarker, circHtra1 needs to be validated using a larger sample size (more than 50 patients in each group). However, as for the chipset study, these preliminary findings with a smaller sample are acceptable like RNA-sequencing data. Second, the effect of circRNAs might be multifaceted. It mostly exerts its role as a miRNA sponge to regulate the target gene expression; meanwhile, it can also directly bind to mRNAs and proteins and sometime translate to peptides as well [17]. Interestingly, circRNA could also directly act as a protein sponge, which is very similar to miRNA function. [24] The circRNA-protein interaction might also be affected by their binding site and tertiary structure, which is very useful for future pharmaceutical design. [17] The multiple mechanisms of circHtra1 need to be further explored in TBI session in future studies as well. Third, the spatial expression patterns of circRNAs in patients with TBI might be different from those in healthy controls; spatial transcriptomics analyses are necessary to address this point. However, poly A enrichment method for spatial transcriptomics are currently not able to investigate the circRNA expression and our results clearly emphasize the role of circRNAs in immune infiltration and suggest that circHtra1 is a candidate biomarker for assessing the neuroimmune response and for predicting outcomes.

## 4. Conclusion

Analyses of the regulatory mechanisms underlying the circRNAs discovered in this study are expected to be valuable for the diagnosis and treatment of TBI. Overall, we obtained a comprehensive circRNA expression profile based on blood samples from patients with TBI. Aberrantly expressed circHtra1 might regulate cell proliferation and the immune response in the injury cascade post-TBI via IGF-1, which is associated with genetic editing, and by sponging miRNAs (Figure S4E). Our results provide novel research directions related to the neuro-endocrine-immune system aimed at the development of effective TBI therapies.

## 5. Materials and Methods

### 5.1. Sample Collection

Peripheral human blood was prospectively obtained and transferred into PAX RNA Tubes (BD, Shanghai, China) within one day post-TBI [25]. Patients with TBI were recruited in 2019 based on brain contusions on initial head CT findings. The study protocol was approved by the local Ethics Committee in Shanghai Pudong New Area People's Hospital (20170223-001 on March 7, 2017). Patients with TBI were classified into groups according to the GCS score: severe group (GCS 3–8), moderate group (GCS 9–12), and mild group (GCS 13–15). Patients who were 18–65 years old with a closed brain injury were included. The exclusion criteria were as follows: (1) severe complication with a thoracic or abdominal injury, (2) serious previous diseases (such as thrombocytopenia and cancer), and (3) family refused to undergo blood collection. Clinical information for patients is listed in Table S1.

### 5.2. Microarray Information

The Agilent Human lncRNA Microarray 2019 (4∗180 k, design ID: 086188) was used in this experiment, and data for the 16 samples were analyzed by OE Biotechnology Co., Ltd. (Shanghai, China).

### 5.3. Gene Microarray

Total RNA was quantified by the NanoDrop ND-2000 (Thermo Scientific, Carlsbad, CA, USA), and the RNA integrity was assessed using the Agilent Bioanalyzer 2100 (Agilent Technologies, Santa Clara, CA, USA). The RNA was manipulated according to the manufacturer's protocols. In brief, total RNA was transcribed to cDNA, synthesized into cRNA, and labeled with Cyanine-3-CTP. The labeled cRNAs were hybridized onto the microarray chipset. After being washed, the chipset was scanned using the Agilent Scanner G2505C (Agilent Technologies).

### 5.4. NK Cells Tested by FACS

To count NK cells in the plasma of patients with TBI, peripheral blood mononuclear cells (PBMCs) were separated from human blood samples. Then, PBMCs were centrifugated at 2000 rpm for 30 minutes at room temperature (23°C). The samples were further tagged with CD3 antibody and fluorescein CD56 antibody (BD Biosciences Pharmingen, CA, USA) for 30 minutes at room temperature. Both monoclonal antibodies were obtained from Invitrogen (CD56 monoclonal antibody, MEM-188 and CD3 monoclonal antibody, OKT3). Human NK cells were gated as CD3-CD56+ lymphocytes for further analysis. FlowJo (Ashland, OR, USA) was used to count the number of NK cells.

### 5.5. Data Analysis

The thresholds for the identification of differentially expressed genes (DEGs) were set at fold change > 2.0 and *P* < 0.05. DEGs were further filtered by a volcano plot. Next, GO and KEGG enrichment analyses of circRNA target genes were performed. GSE data (GSE24047) were used to confirm the differential expression of Htra1 after TBI [26].

### 5.6. ceRNA Network Construction

The miRanda algorithm was used to predict ceRNA interactions. If the expression levels of a circRNA and mRNA were positively correlated, the RNAs were included in the ceRNA analysis. If expressed miRNAs were negatively correlated with both of the above circRNAs and showed complementary binding to both, these miRNAs were identified as competitively inhibited targets of the circRNAs. According to these criteria, the top 200 significant interacting circRNA–mRNA pairs were used to generate a circRNA–mRNA network based on the core circRNAs, and a competing endogenous RNA network was constructed based on the miRNAs shared by the most significant circRNA–mRNA pairs.

### 5.7. sc-RNA-seq Analysis

We applied the 10X Genomics sc-seq in the blood of 12 TBI patients and four healthy controls. The single-cell transcriptome dataset GSE110746 was downloaded from the Gene Expression Omnibus (GEO) database. The chipset data from TBI patients was previously reported and used as an external verification here. R (v. 4.0.2) was used for bioinformatical analysis.

The Seurat package was used for the sc-RNA seq study [13]. The dimension of data was reduced by PCA and t-SNE. Marker genes for different clusters were identified using the Seurat package. All clusters were annotated using the SingleR package with a mouse dataset [14], and cell communication analysis was performed using the Cell-Phone package.

### 5.8. Reagents and Antibodies

The rabbit polyclonal antibody Htra1 (55011-1-AP) and the rabbit polyclonal antibody IGF-1 (PA5-27207) were purchased from Thermo Fisher. Goat *β*-actin antibody (ab8227) and rabbit anti-ADAR1 antibody (bs-2168R; Bioss, Woburn, MA, USA), rabbit polyclonal to GRB10 (ab125583; Abcam, Cambridge, UK), mouse monoclonal to Bcl-2 (ab692; Abcam), and rabbit monoclonal to anti-cleaved caspase-3 (EPR21032; Abcam) were used to evaluate apoptosis in mice with TBI. A mouse monoclonal MAP2 antibody (ab11267; Abcam) and rabbit monoclonal *β*-tubulin antibody (ab201831, Abcam) were utilized to evaluate cell proliferation. AG1024 (121767) was obtained from Sigma-Aldrich (St. Louis, MO, USA), and negative control siRNA (AM4611) was obtained from Invitrogen.

### 5.9. Primary Cortical Neuronal Cultures and Coculture with Astrocytes

Primary cultures of cortical neurons and astrocytes were performed as described previously [10]. Excitotoxicity was introduced by KA treatment of neurons at 1 nmol/L, and the coculture system was described previously [11]. For pharmaceutical intervention, cells were treated with 1 *μ*M IGF-1R inhibitor, si-circHtra1, miR-3960 antagonist, or si-ADAR1. Human embryonic kidney (HEK) 293T cells were cultured in Dulbecco's modified Eagle's medium with d-glucose and 10% fetal bovine serum. For Htra1 overexpression, 3 × 10^6^ HEK 293T cells were transfected with the Htra1-HA plasmid constructed by amplifying genomic cDNA according to the manufacturer's instructions (pcDNA3.1/N-HA vector; Clontech, Oxon, UK).

### 5.10. Mouse TBI Model, qRT-PCR, Western Blotting, and Immunofluorescence Staining

Lateral FPI surgery was performed on 6- to 8-week-old male C57-B6 mice as described previously [10, 27]. The qRT-PCR primers are listed in Table S2. Relative expression levels were calculated with the following formula: 2^–*ΔΔ*Ct^, ΔΔCt = (Ct_target gene_ − Ct_*β*‐actin_) TBI − (Ct_target gene_ − Ct_*β*‐actin_) control. Experiments were repeated at least three times. The relative expression of MAP2 and *β*-tubulin was determined by the average optical density of the fluorescence area. MAP2 is dendritic marker for neurons, while tubulin is an early marker for the maturation of neurons. Therefore, we used these two markers as representative marker for the maturation of neurons.

### 5.11. Plasmid, siRNAs, miRNA Mimic, Inhibitor, Transient Transfection, and Construction of Stable Cell Lines

Plasmid-mediated circRNA overexpression and knockdown vectors were obtained from OE Biotech (Shanghai, China). siRNAs targeting circRNAs were obtained from GenePharma (Suzhou, China), the miR-3960 mimic was obtained from RiboBio (Guangzhou, China), and the lentiviral expression vector for the miR-3960 inhibitor and the control plasmid were obtained from GeneCopoeia (Rockville, MD, USA). For stable transfection, puromycin was used to select cells with stable expression of circHtra1 and the negative vector.

### 5.12. CCK-8 Assay

Each group of cells was adjusted to 1,000 cells per well. Then, 10 mL of CCK-8 solution (Beyotime Biotechnology, Haimen, China) was added to the cell dish after 24 hours. The blank control had only CCK-8 solution. The absorbance (OD) value of each well was read at 490 nm every 24 hours for 3 days.

### 5.13. RIP Assay

The RIP assay was performed using the EZ-Magna RiP Kit (Millipore, Billerica, MA, USA). Cells were lysed in lysis buffer and further incubated with magnetic beads together with human anti-Ago2 (Millipore) or normal human IgG control (Millipore). The IP RNAs were extracted with TRIzol and assessed by qRT-PCR.

### 5.14. Co-IP Assay

For Co-IP of Htra1 and IGF-1, HEK 293T-derived wild-type or Htra1 (1 g) was incubated with human IGF-1 (P5502-1 mg; Beyotime) at 4°C for 2 hours. HtrA1 was immunoprecipitated for overnight incubation. To visualize IGF-1, the bottom half of the PVDF membrane was probed with an IGF-1 monoclonal antibody.

### 5.15. Luciferase Reporter Assay

A luciferase assay was performed as previous reported [28]. Primary cultured neurons (5 × 10^4^ cells per well) were added to a 96-well plate and incubated for 1 day. The related plasmids were transfected using Lipofectamine 3000 (Thermo Fisher Scientific). After 2 days of transfection, luciferase signals were assessed by a luciferase assay (E1980; Promega, Madison, WI, USA). The binding sites of GRB10 and miR-3960 were predicted using RNA22 v2 [29]. In addition, the sequence of circHtra1 containing the putative or mutant putative binding sites for miR-1908-3p, miR-3960, miR-3665-3p, and miR-10400-5p was separately cloned into the pmirGLO vector (Promega). The pmirGLO-circHtra1-WT reporter and pmirGLO-circHtra1-MUT reporter were cotransfected into cells with miRNA mimics, miR-NC, and other miRNA mimics with Lipofectamine 3000. On the third day, a luciferase reporter assay was performed.

### 5.16. HE Staining and PI/Annexin FACS Assay

HE and PI/Annexin staining following the conventional protocol was used to assess the level of apoptosis in the ipsilateral cortex and hippocampus of mice with TBI. All samples were observed under a microscope (Nikon, Tokyo, Japan) and analyzed by FACS (Navios, Beckman Coulter, Brea, CA, USA).

### 5.17. Assessments of Motor Function in Mice with TBI

Motor function was evaluated at 0 (baseline), 1, 3, and 7 days after TBI using the NSS method [30]. Briefly, the test includes forelimb flexion, lateral push, forelimb and hindlimb placement, vestibulomotor function, and motor performance on a balance beam. Neuromuscular functions are scored 0, 1, or 2. Vestibulomotor functions are scored 0–6. Complex neuromotor functions are scored 0–5.

For the paw grasp, grip strength of mice in all groups was evaluated before and after TBI surgery. Neuromuscular function was tested in mice with ipsilateral and contralateral paw grip strength. This was scored by two researchers blinded to the groups on a three-point scale, where one is normal, two is impaired, and three is severely impaired.

### 5.18. Statistical Analysis

All data are presented as means ± standard error of the mean. GraphPad Prism 8.3.1 (USA) was used for statistical analyses. Differences among more than two groups were analyzed by one-way ANOVA and LSD tests. Otherwise, Student's *t*-tests were applied for two-group comparison. Repeated one-way ANOVA was used to analyze the CCK assay results and behavioral assay results. Spearman's correlation analysis was used to assess correlations between two parameters, such as those between GRB10 and NK cell frequencies and GCS. *P* < 0.05 was considered significant.

## Data Availability

The dataset supporting the conclusions of this article are available from the corresponding author.

## Abbreviations

ADAR1: Adenosine deaminase acting on RNA
BP: Biological processes
CC: Cellular components
circRNA: Circular ribonucleic acid
ceRNA: Competitive endogenous RNA
DEGs: Differential expression genes
GCS: Glasgow Coma Scale
GSK3*β*: Glycogen synthesis kinase
GH: Growth factor
HEK: Human embryonic kidney
IGF-I: Insulin-like growth factor 1
IGF-1R: Insulin-like growth factor 1 receptor
KA: Kainic acid
lncRNAs: Long noncoding RNAs
MAPK: Mitogen-activated protein kinase
MS: Mass spectroscopy
MAP: Microtubule-associated protein
MoCA: Montreal Cognition Assessment
METTL1: Methyltransferase-like 1
MF: Molecular functions
MWM: Morris water maze
NK: Natural killer
PBMCs: Peripheral blood mononuclear cells
MTT: Thiazolyl blue tetrazolium bromide
TBI: Traumatic brain injury
WT: Wildtype.

## Supplementary Materials

Supplementary Materials Figure S1: the ceRNA network for circular RNA-miRNA-mRNA. Figure S2: treatment with si-circHtra1 significantly prevents the TBI-associated injury and motor deficits. Figure S3: quantitative PCR of circHtra1 and Htra1 level in whole blood from TBI patients. Figure S4: the cellular expression of Grb10 and Htra1 level in the ADAR1 KO cell line. Table S1: clinical characteristics of patients with traumatic brain injury. Table S2: list of all primer sequences used in real-time PCR experiment. Table S3: top five upregulated and downregulated circRNAs in severe TBI.
