# Supplementary material for: circHtra1/miR-3960/GRB10 Axis Promotes Neuronal Loss and Immune Deficiency in Traumatic Brain Injury
Source: Oxid Med Cell Longev. 2022 May 6;2022:3522492. doi: 10.1155/2022/3522492 (PMC9106453; doi:10.1155/2022/3522492)
Supplement: Supplementary Materials — Figure S1: the ceRNA network for circular RNA-miRNA-mRNA. Figure S2: treatment with si-circHtra1 significantly prevents the TBI-associated injury and motor deficits. Figure S3: quantitative PCR of circHtra1 and Htra1 level in whole blood from TBI patients. Figure S4: the cellular expression of Grb10 and Htra1 level in the ADAR1 KO cell line. Table S1: clinical characteristics of patients with traumatic brain injury. Table S2: list of all primer sequences used in real-time PCR experiment. Table S3: top five upregulated and downregulated circRNAs in severe TBI. [file 3522492.f1.docx]

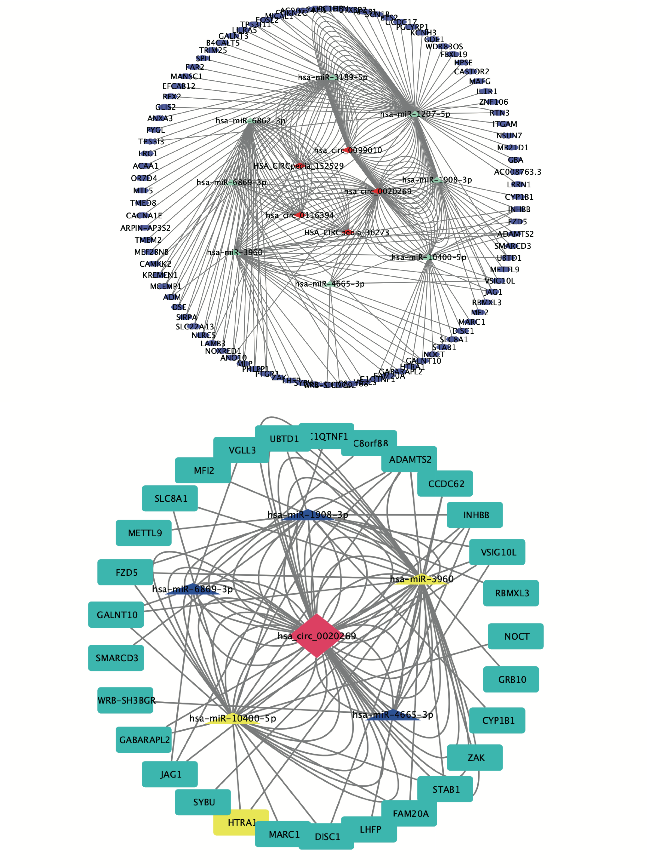

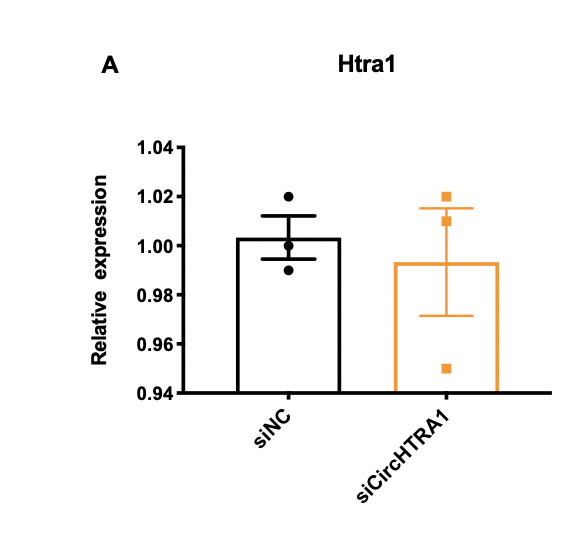


**C**


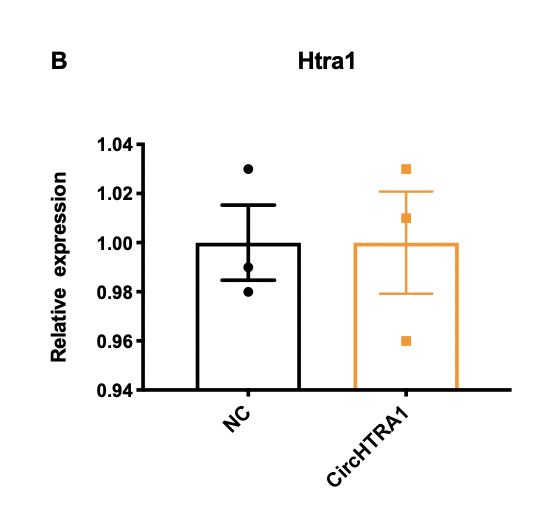

**Figure S1. ceRNA network for circular RNA-miRNA-mRNA.** (A&B) mRNA levels of *Htra1* in neurons treated with si-circHtra1 (t=0.4243, df=4) or a circHtra1 (t=0.0000, df=4) overexpression vector. (C) The network chooses 200 best co-expression pairs for potential competitive endogenous RNA analysis. The miRNAs targeted by the five top circRNA-mRNA pairs based on the network were analyzed, and the circRNA-miRNA-mRNA network was constructed. Besides 5 circRNAs (yellow squares) and 8 miRNAs (red dots), there are 98 blue squares representing the mRNAs. The top five miRNAs that potentially regulate each pair of circRNA-mRNA are labeled with green enlarged squares. Color image is available online. The circular ribonucleic acid (RNA) microRNA-messenger RNA (circRNA_0020269-miRNA-mRNA) interaction network. The circRNA_0020269 was labeled by red diamond, miRNAs were labeled by blue triangle, and green notes are mRNAs. Htra1 was highlighted using an enlarged yellow note. Color image is available online. (D) Early apoptosis quantification in each group (t=8.419, 11.99, 29.22, df=4, respectively); (E) Late apoptosis quantification in each group (t=24.52, 21.48 & 0.7998, respectively); Compared with the PBS group, **P* < 0.05; compared with the si-NC group, #*P* < 0.05; compared with the siRNA+miR antagomir group, &*P* < 0.05.


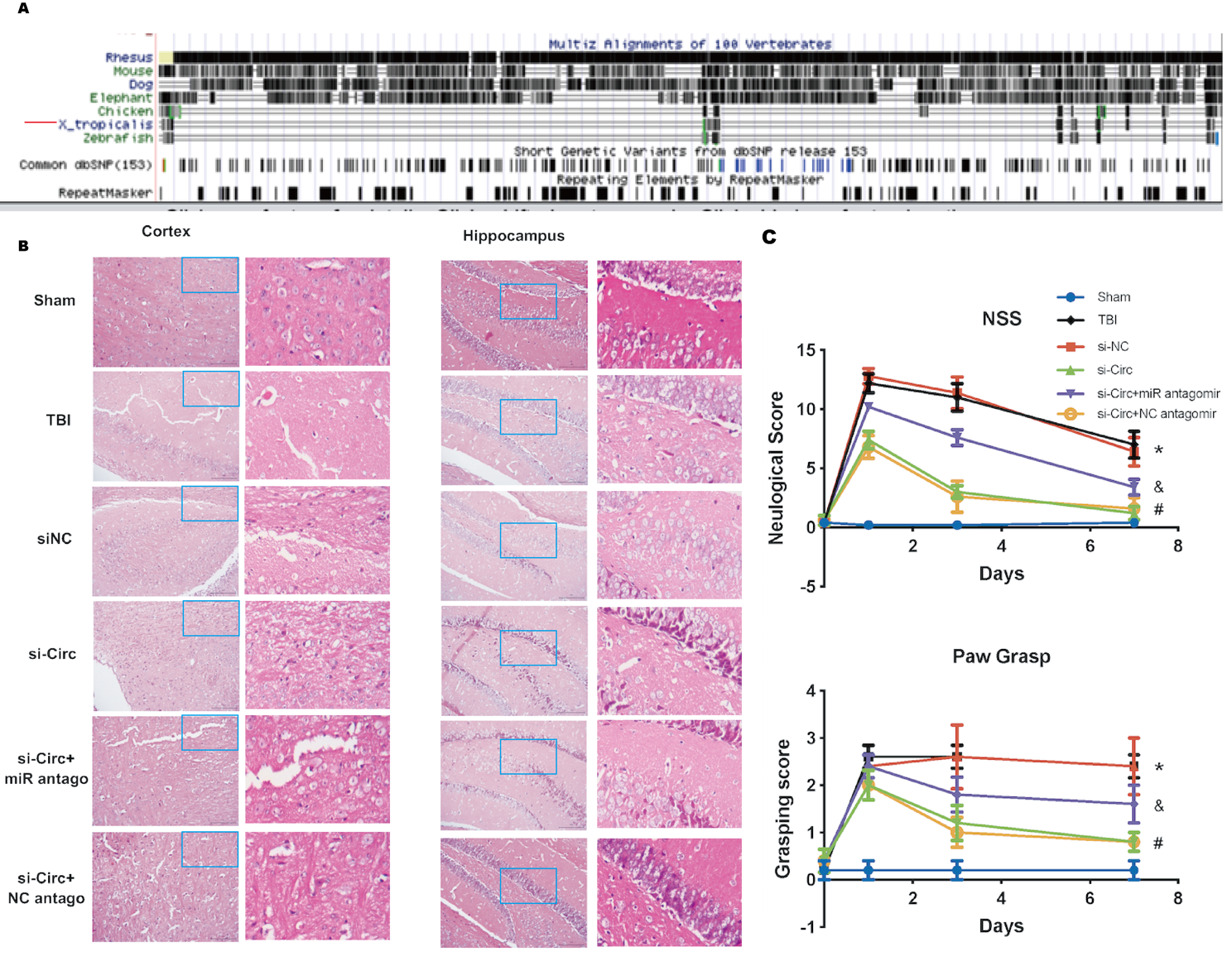


Figure S2. **Treatment with si-CircHtra1 significantly prevents the TBI-associated injury and motor deficits. (A)** UCSC Genome Browser on Human Assembly UCSC Genome Browser on Human Assembly (GRCh37/hg19) and searched the chr10:124221040-124274424 which includes 53385 bp, we found the circHtra1 was conserved in Rhesus, Mouse, Dog and Elephant. Therefore, we applied mouse model for further in-vivo research. **(B)** Compared to the sham group, TBI group showed edema around neuronal cells with decreased staining of cell nucleus, enlarged microvascular space and cavity; while si-Circ treatment could reduce the microvascular space and neuronal death in both ipsilateral cortex and hippocampus and this effect was partly abolished by treatment together with miR-3960 antagonist. In addition, graphs show motor assessment using NSS and paw grasp test **(C).** Repeated one-way ANOVA demonstrated significant effects as follows: NSS, treatment effects F(4,80) = 64.67; paw grasp treatment effect F(4,80) = 23.27. LSD tests are used to compare between sham and TBI, *p < 0.01; between TBI+si-Circ and TBI, # p < 0.01; comparison between TBI+si-Circ+miR antagonist and TBI+si-Circ, & p < 0.01.

**Figure S3**. **Quantitative PCR of circHtra1 and Htra1 level in whole blood from TBI patients.** (A) The 10 up-regulated circRNAs in severe TBI. (B) Expression levels of circ-Htra1 in different groups of TBI. (C) Expression of Htra1 in different groups of TBI F(3,12)=9.591. (D) GDS data shows the expression of Htra1 in a rat model of TBI. (E) Quantification of circ-Htra1 in different groups of TBI, which is consistent with microarray data, F(3,12)=19.09. (F) Correlation between circHtra1 and Htra1 expression (P=0.01). The correlation between the level of circRNAs and GCS with Pearson’s analysis.


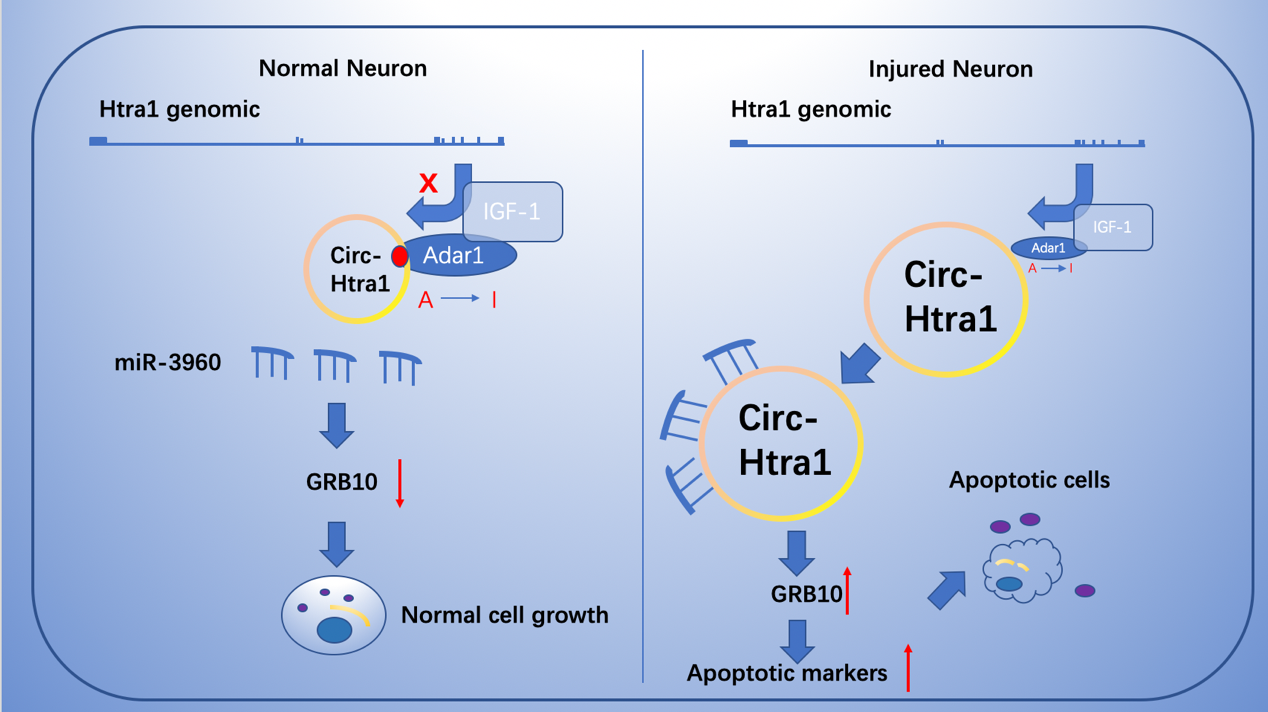

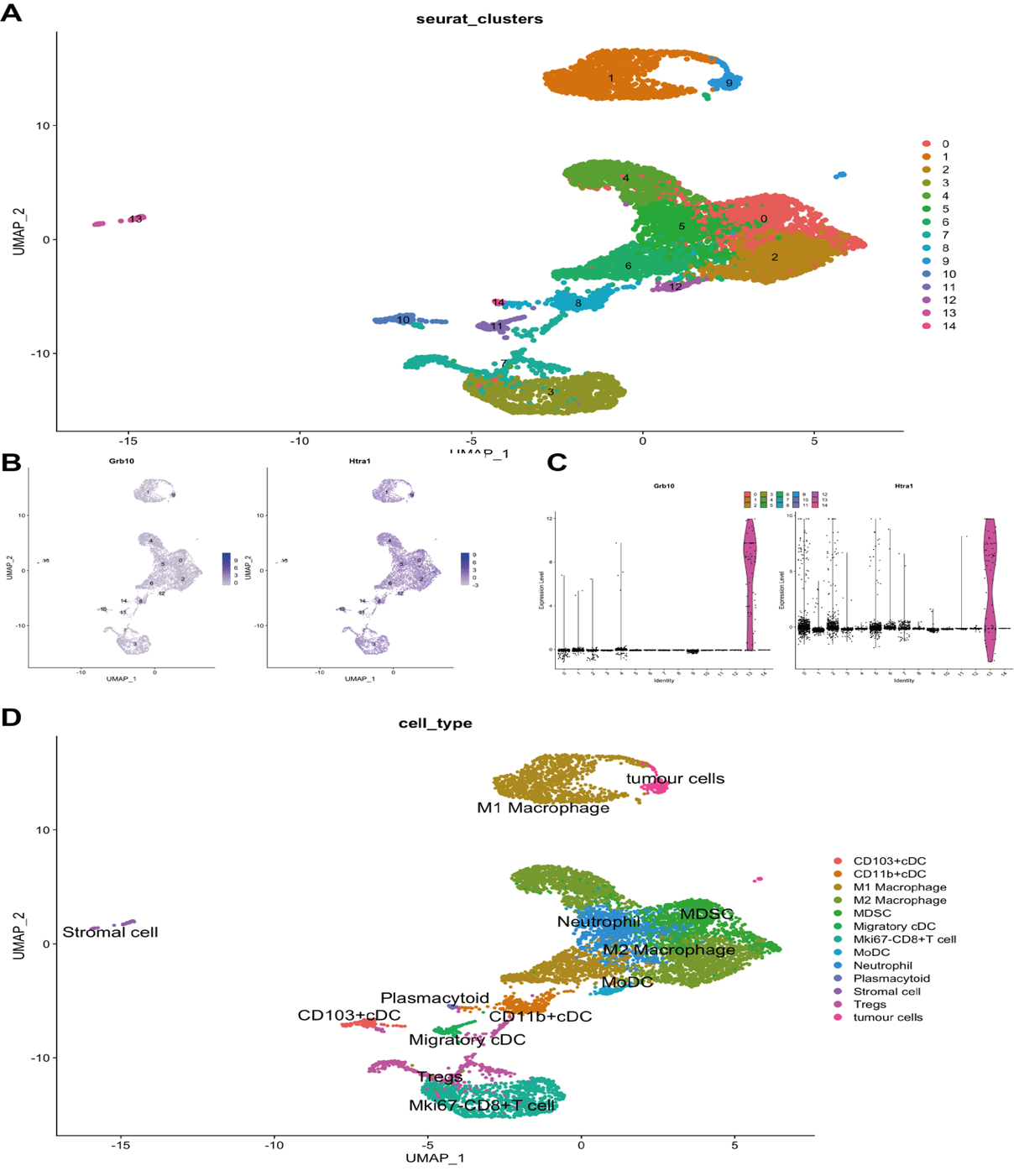


**E**

**Figure S4. The cellular expression of Grb10 and Htra1 level in ADAR1 KO cell line.** (A) The umap of sc-seq data in ADAR1 KO cells. (B & C) Dominant expression of Grb10 and Htra1 in Stromal cells. (D) Cell annotation of 15 clusters in ADAR1 KO cells. (E) **Schematic representation of the function and mechanism of circ-Htra1 in primary cultured neurons.** CircHtra1 level is significantly increased in injured neurons. CircHtra1 acts as a miR-3960 sponge to regulate the miR-3960/GRB10 in neurons. Injured neurons have increased circ-Htra1 expression levels due to lower ADAR1 in neurons, which further decreases the activity of miR-3960; therefore, upregulated Htra1 expression ultimately promotes apoptosis in neurons.

**Table S1 Clinical characteristics of patients with traumatic brain injury**

| Patient | Severity | Age(year) | Sex | Brain injury | GCS | GCS(EVM) |
| --- | --- | --- | --- | --- | --- | --- |
| 1. Mi 1 | Mild | 48 | F | Laceration | 14 | E3V5M6 |
| 1. Mi 2 | Mild | 28 | M | Contusion | 15 | E4V5M6 |
| 1. Mi3 | Mild | 41 | M | SAH | 15 | E4V5M6 |
| 1. Mi4 | Mild | 47 | F | SDH | 15 | E4V5M6 |
| 1. Mo1 | Moderate | 29 | F | Laceration | 12 | E3V4M5 |
| 1. Mo2 | Moderate | 49 | F | SDH | 12 | E3V3M6 |
| 1. Mo3 | Moderate | 29 | M | SAH | 12 | E3V4M5 |
| 1. Mo4 | Moderate | 50 | M | SAH | 12 | E2V4M6 |
| 1. Se1 | Severe | 23 | M | Brain stem injury | 3 | E1V1M1 |
| 1. Se2 | Severe | 43 | F | Contusion | 8 | E2V2M4 |
| 1. Se3 | Severe | 44 | F | SDH | 6 | E1V1M4 |
| 1. Se4 | Severe | 60 | M | SDH | 6 | E1V1M4 |
| 1. Co1 | Control | 40 | M |  |  |  |
| 1. Co2 | Control | 46 | F |  |  |  |
| 1. Co3 | Control | 50 | F |  |  |  |
| 1. Co4 | Control | 45 | M |  |  |  |

SAH：subarachnoid hemorrhage; SDH: subdural hemorrhage; F: Female, M: Male.

**Table S2. List of all primer sequences used in real-time PCR experiment.**

| **Gene** | Forward primer | Reverse primer |
| --- | --- | --- |
| **Htra1** | 5ʹ-CCAAAGAGCTGAAGGACCGT-3ʹ | 5ʹ-TGACCACAGACTGTCCGTTG-3 |
| **circHtra1** | 5ʹ-ATGACTCTGAGTTTGAGCTA-3ʹ | 5ʹ-TGCCAATGGGCTGGGCCGCG-3ʹ |
| **GRB10** | 5ʹ-GTGAACCCTTTCCAAACGAC-3ʹ | 5ʹ-GTGTGGCGAAGTCTGTGCTA-3ʹ |
| **GAPDH** | 5ʹ-ATGACTCTACCCACGGCAAG-3ʹ | 5ʹ-CTGGAAGATGGTGATGGGTT-3ʹ |
| **18S rRNA** | 5ʹ-CATTCGAACGTCTGCCCTAT-3ʹ | 5ʹ-GTTTCTCAGGCTCCCTCTCC-3ʹ |
| **U6** | 5ʹ-TGCTTCGGCAGCACATATAC-3ʹ | 5ʹ-AGGGGCCATGCTAATCTTCT-3ʹ |

**Table S3. Top five up-regulated and down-regulated circRNAs in severe TBI**

| CircRNA | P Value | FC | Log2FC | Chromosome | Strand |  |
| --- | --- | --- | --- | --- | --- | --- |
| Top five up-regulated circRNAs | | | | | | |
| hsa_circ_0020273 | 1.43E-05 | 19.3401702 | 4.27352859 | chr10 | + |  |
| hsa_circ_0093014 | 1.07E-05 | 18.1460844 | 4.18158637 | chr10 | + |  |
| HSA_CIRCpedia_152345 | 0.00086533 | 17.4714387 | 4.12692651 | chr16 | + |  |
| hsa_circ_0020269 | 1.59E-05 | 17.1563439 | 4.10067023 | chr10 | + |  |
| hsa_circ_0064339 | 0.00026419 | 12.5882379 | 3.65400444 | chr3 | + |  |
| Top five down-regulated circRNAs | | | | | | |
| hsa_circ_0116097_10_18 | 0.00438551 | -8.8794837 | -3.1504758 | chr21 | + |  |
| hsa_circ_0070423_0_21 | 0.00833559 | -9.8995302 | -3.3073601 | chr4 | + |  |
| HSA_CIRCpedia_35578_7_22 | 0.00252167 | -9.9066783 | -3.3084014 | chr21 | + |  |
| hsa_circ_0129469_16_1014 | 0.00961041 | -11.449644 | -3.5172309 | chr5 | + |  |
| hsa_circ_0061796_16_32 | 0.00100071 | -14.620232 | -3.8698943 | chr21 | + |  |
